# Crosstalk of MicroRNAs and Oxidative Stress in the Pathogenesis of Cancer

**DOI:** 10.1155/2020/2415324

**Published:** 2020-04-28

**Authors:** Can Lu, Danting Zhou, Qiang Wang, Wenliang Liu, Fenglei Yu, Fang Wu, Chen Chen

**Affiliations:** ^1^Centre of Stomatology, Xiangya Hospital, Central South University, Changsha 410008, China; ^2^Department of Thoracic Surgery, The Second Xiangya Hospital, Central South University, Changsha, Hunan 410011, China; ^3^Department of Oncology, The Second Xiangya Hospital, Central South University, Changsha, Hunan 410011, China

## Abstract

Oxidative stress refers to an imbalance between reactive oxygen species (ROS) generation and body's capability to detoxify the reactive mediators or to fix the relating damage. MicroRNAs are considered to be important mediators that play essential roles in the regulation of diverse aspects of carcinogenesis. Growing studies have demonstrated that the ROS can regulate microRNA biogenesis and expression mainly through modulating biogenesis course, transcription factors, and epigenetic changes. On the other hand, microRNAs may in turn modulate the redox signaling pathways, altering their integrity, stability, and functionality, thus contributing to the pathogenesis of multiple diseases. Both ROS and microRNAs have been identified to be important regulators and potential therapeutic targets in cancers. However, the information about the interplay between oxidative stress and microRNA regulation is still limited. The present review is aimed at summarizing the current understanding of molecular crosstalk between microRNAs and the generation of ROS in the pathogenesis of cancer.

## 1. Introduction

Reactive oxygen species (ROS), mainly including hydroxyl radicals (HO^·^), superoxide (O_2_^·-^), and hydrogen peroxide (H_2_O_2_), are usually generated under physiological conditions and have essential functions in living organisms [1–3]. Usually, a moderate increase level of ROS can promote cell differentiation and proliferation, whereas overproduction of ROS may result in oxidative damage to lipids, DNA, and proteins [4]. Therefore, maintaining ROS homeostasis has a crucial role for normal cell growth and survival. Generally, the cellular levels of ROS are cautiously monitored by the natural antioxidant defense network so that the redox homeostasis could be maintained. Disruption of normal redox state (a condition termed as oxidative stress), either because of excessive amounts of ROS or dysfunction of antioxidant defense system, would result in toxic damages through the production of free radicals and peroxides, thus give rise to pathophysiological situation that lead to multiple diseases, including cancer [3, 5].

Compared to normal cells, cancer cells usually have elevated levels of ROS, reflecting a disturbance in redox hemostasis. This may attribute to enhanced metabolic activity and disrupted cellular signaling [1, 6, 7]. It is believed that ROS changes in cancer cells are very complicated due to the multiple factors that modulate the ROS hemostasis and stress response [1, 8, 9]. Under persistent oxidative stress circumstances, cancer cells may evolve a particular set of adaptive mechanisms, which not only enhance ROS scavenging systems to deal with the stress but also suppress cell apoptosis. On the other hand, cancer cells with increased ROS level are more likely to be susceptible to damage due to excessive amounts of exogenous agents [10, 11]. Several studies have indicated that, to efficiently kill cancer cells and reduce drug resistance related to oxidative damage, it is pivotal to understand the complicated ROS alternations in cancer cells and the underlying regulation mechanisms [12].

MicroRNAs are small noncoding RNA molecules with a length of 19 to 25 nt that play an essential role in posttranscriptional regulation by binding to the 3′-untranslated regions (UTRs) of target gene and regulating various cellular processes, such as cell proliferation, apoptosis, and stress response [13, 14]. Previous studies have found that the regulation of microRNAs was cell type- and tissue-specific. A single microRNA may target various mRNAs, meanwhile a single mRNA could be modulated by multiple microRNAs. Therefore, microRNAs can regulate different groups of mRNAs involved in diverse pathological conditions and the pathogenesis of various human diseases, such as immune system disorders and malignancies [15]. On the other hand, the relative stability of microRNA made it has the possibility to be novel diagnostic biomarkers and potential therapeutic targets for various types of cancers [16].

Mounting evidence from previous studies has implied that the expression of microRNA altered in response to ROS accumulation [17]. Besides the ROS-mediated tumor progression, it is believed that ROS production also plays a vital role in the microRNA-related mechanisms of cancer development. It is principal to understand the interplay between ROS production and microRNAs in carcinogenesis, since both of them have been demonstrated to be dysregulated and have great potential to be novel therapeutic targets in cancer. The present review focused on the comprehensive summarization of the current understanding of molecular crosstalk between ROS production and microRNAs in the pathogenesis of cancer.

## 2. MicroRNAs Modulate ROS Production through Targeting Multiple Signaling Pathways

Several studies have revealed that ROS production could be modulated by microRNAs through regulating several redox signaling pathways. By targeting the Nrf2/Keap1 pathway, SOD/catalase signaling pathway, mitochondrial regulatory pathway, and several essential enzymes, microRNAs could regulate intracellular redox hemostasis and affect the carcinogenic processes (Table 1).

### 2.1. Nrf2/Keap1 Signaling Pathway

It has been characterized that the transcription factor nuclear factor erythroid-derived 2-like 2 (Nrf2) and its inhibitor Kelch-like ECH-associated protein 1 (Keap1) are crucial regulators in body's response to oxidative stress [18, 19]. Under oxidative stress, Nrf2/Keap1 complexes degenerate and Nrf2 is transferred to the nucleus. This alternation enhances the expression and activity of several antioxidant genes that inhibit cell apoptosis, at the same time promote cancer cell survival and tumorigenesis [20, 21]. Previous studies have revealed that the Nrf2 signaling pathways were targeted by multiple microRNAs, including miR-144, miR-28, miR-200a, and miR-93. Karihtala and colleagues observed that upregulation of miR-93 in pancreatic cancer was negatively associated with Nrf2 expression and predicted better cell differentiation [22]. On the other hand, Singh et al. found that in breast cancer, miR-93 could decrease multiple carcinogenic processes in an Nrf2-dependent manner. Silencing of miR-93 could promote cell apoptosis and inhibit colony formation, mammosphere formation, and cell migration [23]. Another example of the Nrf2/Keap1 pathway-targeted microRNA is miR-200a. Eades et al. and Yang et al. reported the miR-200a and miR-28 could regulate Nrf2 expression via directly targeting Keap1 mRNA in breast cancer cells [24, 25].

Moreover, Gu and colleagues reported that the upregulation of miR-155 in lung cancer cells could promote tumor cell colony formation and migration, as well as repressing cell death. This was mediated by upregulating the Nrf2/Keap1 signaling pathway. Downregulation of miR-155 would significantly decrease the cellular levels of Nrf2, NAD(P)H quinone oxidoreductase 1, and heme oxygenase-1 (HO-1), thus suppressing cancer cell survival and migration and facilitating cell apoptosis [26]. Another important Nrf2 regulating microRNA is miR-125b. Chen and colleagues reported that miR-125b was the upregulator of peroxiredoxin-like 2A (PRXL2A), an antioxidant molecule that protects cells from oxidative damage. The Nrf2 was then identified to be a downstream effector of the miR-125b-PRXL2A axis [27] (Table 1).

### 2.2. Mitochondrial Signaling Pathway

Mitochondria are considered to be one of the primary sources of ROS [28, 29]. To maintain normal cellular processes, mitochondria undergo fission and fusion continually and generate a proper level of ROS in response to alterations in the surrounding circumstances. However, oxidative stress may lead to abnormal mitochondrial dynamics and dysfunction of related signal pathways [29]. MicroRNAs could regulate the activity and expression of several mitochondrial proteins, which involved in the maintaining of redox homeostasis. For instance, it has been revealed that upregulated miR-210 could increase the ROS level in cancer cells by suppressing the iron-sulfur cluster recruiting proteins ISCU1/2 [30, 31]. Mitochondrial dysfunction induced by miR-210 would upregulate the glycolysis rate and make tumor cells more susceptible to glycolysis inhibitors [30]. Overexpression of miR-210 could also induce ROS generation under hypoxic condition, indicating worse prognosis in colorectal cancer [31]. Additionally, Tagscherer and colleagues reported that miR-210 could induce cancer cell apoptosis via promoting ROS generation [32]. Another microRNA, miR-34a, has been found to repress ROS generation by downregulating the genes that code for ROS-related enzymes and mitochondrial complexes, resulting in apoptosis resistance. Restoration of miR-34a could sensitize the tumor cell in response to oxidative stress [33].

Muys and colleagues reported that miR-450a could act as a tumor suppressor by inhibiting glycolysis and glutaminolysis via targeting a set of mitochondrial related genes, such as ACO2, TIMMDC1, ATP5B, and MT-ND2. Overexpression of miR-450a decreased mitochondrial membrane potential but increased glucose uptake and viability, featured of less invasive cancer cell lines [34, 35]. Kao and colleagues observed that miR-31 could target SIRT3 to suppress mitochondrial activity and enhance oxidative stress in oral cancer [36]. Fan et al. revealed the mitochondrial microRNAs (mitomiR) mitomiR-2392 regulated chemoresistance in tongue squamous cell carcinoma by downregulating oxidative phosphorylation and upregulating the glycolysis [37]. Xu and colleagues reported miR-17-3p could repress three major mitochondrial antioxidant enzymes (manganese superoxide dismutase (Mn-SOD), glutathione peroxidase 2 (Gpx2), and thioredoxin reductase 2 (TrxR2)) and enhance the radiosensitivity of prostate cancer cells [38]. These studies pose a profound impact on the regulatory role of microRNAs on the function and protein synthesis of the mitochondrial regulatory pathway (Table 1).

### 2.3. SOD/Catalase Signaling Pathway

Superoxide dismutases (SOD), including Fe/Mn-SOD, Cu/Zn-SOD, and Ni-SOD family members, are metal ion cofactor-requiring enzymes that catalyze the dismutation of O_2_^·-^ into O_2_ and H_2_O_2_. Different from the Nrf2/Keap1 system, SOD/catalase system acts out their antioxidant functions by promoting specific biochemical reactions to remove accumulated ROS [39]. It has been reported that increased expression of Mn-SOD could be detected in gastric cancer and esophageal squamous cell carcinoma [39–41]. Colorectal malignancy is also correlated with enhanced Mn-SOD activity and expression. On the other hand, Cu/Zn-SOD is downregulated in cancer tissues than that in normal tissues [42]. Most of the SODs could be targeted by microRNAs as a regulating mechanism underlying their antioxidant functions.

miR-21 has been proved to play an essential role in several aspects of tumorigenesis. It has been indicated that miR-21 could promote carcinogenesis through suppression of SOD2 or SOD3 by targeting TNF-*α* generation, thus suppressing the dismutation of superoxide to the less damaging molecule of H_2_O_2_ [43–46]. Wang et al. observed that miR-146a could regulate the catalase mRNA to degeneration. Silencing of miR-146a has been reported to improve the antioxidant ability in cisplatin-treated lung cancer cells through increasing catalase level, which was the main reason leading to drug resistance [47]. Patel and colleagues reported that exosome-delivered miR-155 could increase the level of SOD2 and catalase through inhibiting a gemcitabine metabolizing enzyme, DCK, causing chemoresistance in pancreatic cancer cells [48]. Besides, Meng and colleagues investigated that miR-212 could directly target and downregulate Mn-SOD mRNA expression, thereby suppressing Mn-SOD-induced colorectal cancer metastasis [49] (Table 1).

## 3. ROS Regulates the Expression and Biogenesis of MicroRNAs

Emerging evidence indicated that ROS regulated several facets of microRNA transcription, maturation, and function. ROS could directly modulate the activity of vital proteins that control posttranscriptional events in the biogenesis of microRNAs. On the other hand, a certain group of transcription factors was upregulated under oxidative stress and directly activated the transcription of a subset of microRNAs. Also, ROS has been directly implicated in epigenetic alternations such as DNA methylation and histone modifications that control specific microRNA transcription [50]. Together, these findings indicated ROS as an important proximal regulator of microRNA biogenesis and function (Table 2).

### 3.1. DGCR8 and Dicer

Elevating ROS level by increased oxidative stress has been shown to induce abnormal expression of a particular set of microRNAs [51]. For example, the exposure of exogenous H_2_O_2_ could result in the upexpression of miR-21, as well as downregulated miR-27, miR-29b, and miR-328, indicating these microRNAs are redox-sensitive and functional relating to ROS hemostasis [8, 17]. Moreover, ROS generation also participates in the processing of pre-microRNA transcripts to mature microRNAs. The RNA-binding protein DiGeorge critical region-8 (DGCR8) is a key regulator of maturation of canonical microRNAs. The deletion of DGCR8 would lead to a deficiency in producing all canonical microRNAs [52]. Several studies have revealed that intracellular redox hemostasis could directly modulate DGCR8 activity, therefore regulating microRNA biogenesis [52–54].

Dicer, another pivotal protein of the microRNA biogenesis machinery that is responsible for the synthesis of mature microRNAs, has been identified to be suppressed by oxidative stress through modulating the Nrf2/Keap1/ARE pathway [55, 56]. Increased activity of Nrf2 could upregulate the expression of Dicer protein. Cheng and colleagues demonstrated an ARE consensus sequence in the 5′ flanking region of the Dicer genes, indicating the Nrf2/Keap1/ARE pathway might be an essential regulator of microRNA synthesis [57]. Moreover, the let-7 microRNA family has been found to overexpress under oxidative stress environment. Since let-7 microRNA family could directly suppress Dicer genes, it has been considered as a potential regulating mechanism accounting for decreased Dicer expression and subsequent microRNA-related redox imbalance [58].

### 3.2. Transcription Factors

It is of note that the expression of microRNAs is regulated by several transcription factors, such as nuclear factor *κ*B (NF-*κ*B), c-Myc, and HIF-1*α* (Table 2). A growing number of studies have proved that some of these transcription factors were ROS-sensitive, suggesting aberrant microRNA expression in tumorigenesis was closely related to oxidative stress. Some microRNAs such as miR-19a, miR-29, miR-31, and miR-155 have been demonstrated to be regulated by NF-*κ*B, which could be activated or inactivated by ROS [8, 59]. TGF*β*1-mediated ROS production could facilitate the NF-*κ*B nuclear translocation and subsequently upregulate miR-146a and miR-21 in the development of lung cancer, AML, and colorectal cancer [60–62]. On the other hand, TNF*α*-induced oxidative stress could suppress NF-*κ*B activity and the expression of its target miR-19a and miR-155 [63, 64]. Additionally, Cheleschi and colleagues reported that miR-34a and miR-181a could mediate cell apoptosis and oxidative stress via targeting NF-*κ*B pathway [59]. These studies indicated a comprehensive ROS-NF-*κ*B-microRNA transcription regulatory system.

It has been reported that HIF-1*α*, an important ROS-responsive transcription factor, could activate the transcription of miR-210 by directly binding to its promoter region, resulting in cell differentiation [65]. Moreover, a set of microRNAs (such as miR-135, miR-421, miR-382, and miR-687), which were abnormally expressed during tumor growth and metastasis, have also been reported to be regulated by HIF-1*α* [66–68]. Additionally, C-Myc, which is also ROS-sensitive, could either upregulate the expression of oncogenic microRNAs such as miR-17, or suppressing the expression of some tumor suppressor microRNAs such as miR-34a, miR-137, and miR-15a, therefore promoting cancer development [69, 70]. Sun and colleagues observed the regulating status of the C-Myc-miR-137-EZH2 pathway in ovarian cancer. Activated C-Myc-miR-137-EZH2 axis was observed in chemoresistant ovarian cancer cells and was sustained by elevated ROS production [71]. Li and colleagues investigated that by regulating miR-17-92, MYC could maintain a neoplastic state through suppressing a certain set of chromatin regulatory genes, such as Hbp1, Sin3b, and Btg1, as well as the apoptosis regulator gene Bim, thereby regulating the cell survival, proliferation, and apoptosis [72]. The emerging concept of the TF-ROS-microRNA regulatory network provided important perspectives on the complexity of microRNA function and regulation during carcinogenesis.

### 3.3. Epigenetic Alternations

Another important regulating mechanism that needs to be emphasized is epigenetic alterations. There is no doubt that either DNA methylation or histone modifications are closely related to various environmental stresses, especially oxidative stress. DNA methyltransferases (DNMTs) and histone deacetylases (HDACs) are two major enzyme families that play essential roles in the epigenetic regulation of gene transcription, including microRNAs. The expressions and activities of DNMTs and HDACs can be regulated by oxidative stress and therefore represents a crucial regulating mechanism by which ROS can control microRNA expression and biogenesis (Table 2). For example, HDAC4 could be overexpressed in cancer cells under oxidative stress, which allows for miR-1 and miR-206 promoter deacetylation and lower gene expression, leading to lung cancer progression [73]. SAHA, a histone deacetylase inhibitor, could induce apoptosis in lung cancer cells via upregulating miR-129-5p [74]. Zhang and colleagues observed the epigenetic regulation of miR-29 by targeting HDAC3, MYC, and EZH2 in lymphomas. They found that MYC suppressed miR-29 expression via forming a corepressor complex with HDAC3 and EZH2. By inhibiting miR-26a expression, MYC contributes to EZH2 upregulation. On the other hand, EZH2 could induce MYC expression through downregulating miR-494, thus forming a positive feedback. Suppression of HDAC3 and EZH2 cooperatively inhibited the MYC-EZH2-miR-29 axis, leading to reexpression of miR-29, therefore downregulating miR-29-targeted genes and inhibiting lymphoma progression [75].

## 4. The Crosstalk between ROS and MicroRNAs in Carcinogenesis

Previous studies have shown that ROS can directly regulate microRNA expression and biogenesis. On the other hand, microRNAs may in turn modulate the redox signaling pathways, altering their integrity, stability, and functionality, thereby contributing to the pathogenesis of diverse disease processes, especially cancers. These comprehensive regulatory networks have been observed in several important hallmarks during carcinogenesis, such as cell growth, proliferation, apoptosis, EMT process, and metastasis.

### 4.1. Cell Growth and Proliferation

Both ROS and microRNAs have been identified to regulate cell growth and proliferation. The Nrf2/Keap1 pathway was shown to be the crucial regulators of endothelial glycolysis and cell proliferation with miR-93 and mediate the effects of oxidized phospholipids on endothelial activation [76]. Activation of KRAS could induce upregulation of miR-155 in pancreatic cancer cells. By inhibiting FOXO3a, overexpressed miR-155 could decrease major antioxidants including SOD2 and catalase and stimulate cell proliferation [77]. He and Jiang revealed that upregulation of miR-200a and miR-141 could modulate ROS production under oxidative stress by targeting p38a and promote cell growth and proliferation [17]. Another example involved miR-192-5p, which could be upregulated by H_2_O_2_ exposure in a p53-dependent manner. Downregulated miR-192-5p could affect multiple cellular processes, including cell cycle, DNA repair, and stress response. Hence, overexpression of miR-192-5p significantly reduced endothelial cell proliferation and cell death [78]. Moreover, Degli Esposti and colleagues found that miR-500a-5p could directly regulate several oxidative stress-related genes. Exposure to H_2_O_2_ induced overexpression of miR-500a-5p and decreased the transcription of oxidative stress-related genes NFE2L2 and TXNRD1. Overexpression of TXNRD1 was found to be associated with ER+breast cancer prognosis. Thus, their study identified miR-500a-5p as an oxidative stress response microRNA whose expression might be closely related to cancer progression and survival [79].

### 4.2. Cell Death and Apoptosis

Programmed cell death, or apoptosis, is usually featured by distinct morphological features and energy-dependent biochemical mechanisms. Apoptosis is believed to be a crucial part of multiple cellular processes including cell turnover, atrophy, and necroptosis [80, 81]. ROS plays an essential role in the regulation of numerous cell death-related signaling pathways, such as the RAS/MAPK and/or JNK signaling pathways [82, 83]. Wu et al. found that ginsenoside Rh4 (RH4) could induce cell apoptosis and autophagy via activating the ROS/JNK/p53 pathway in colorectal cancer cells, indicating the Rh4 has great potential to be an anticancer agent [84]. Mohammad and colleagues demonstrated that JNK signaling could activate the Nrf2/Keap1 pathway and at the same time contribute to piperlongumine-induced cell death in pancreatic cancer cells [85].

Moreover, Yan and colleagues found miR-762 participated in the regulation of mitochondrial function by targeting NADH dehydrogenase subunit 2, thereby regulating cell apoptosis [86]. Also, downregulation of miR-101-3p caused elevated Bim expression, which would activate the intrinsic pathway of apoptosis via interacting with Bcl-2, thereby decreasing mitochondrial membrane potential, ROS production, and caspase activation [87]. Pant and colleagues reported that butyrate, one of the short-chain fatty acids generated by the gut microbiota during anaerobic fermentation of dietary fibers, could induce ROS-mediated apoptosis by regulating the miR-22/SIRT-1 pathway in hepatocellular carcinoma [88]. These studies implied that microRNAs could regulate programmed cell death or apoptosis through targeting multiple signaling pathways that related to ROS production.

Ferroptosis, an iron-dependent modulated form of necrosis, was recently recognized as a critical regulating mechanism of cell death driven by ROS accumulation [89]. Iron and iron-catalyzed oxidative stress have caused much interest due to their complex regulation of cellular signaling related to cell death and apoptosis [90]. Luo and colleagues showed that miR-137 negatively regulated ferroptosis by directly targeting glutamine transporter SLC1A5 in melanoma [91]. Xiao and colleagues observed that overexpression of miR-17-92 significantly decreased erastin-induced growth inhibition and ROS production in endothelial cells. Further studies indicated that erastin-induced ferroptosis was correlated with GPX4 downregulation and ACSL4 overexpression. miR-17-92 could directly target the A20-ACSL4 axis, thus protecting endothelial cells from ferroptosis [92]. Additionally, Zhang and colleagues reported that upregulation of miR-9 inhibited glutamic-oxaloacetic transaminase GOT1 by directly binding to its 3′-UTR, which subsequently decreased erastin-and RSL3-induced ferroptosis [93].

### 4.3. Epithelial-Mesenchymal Transition, Tumor Invasion, and Metastasis

Epithelial-mesenchymal transition (EMT), a process in which epithelial cells acquire mesenchymal features, has been identified in multiple pathophysiological conditions, including organ fibrosis and cancer invasion and metastasis [94, 95]. Dysregulation of ROS and microRNAs is involved in the EMT process, thus affecting the tumor invasion and metastasis. Several microRNAs (such as miR-200 family) can directly regulate the EMT process in either normal cells or tumor cells. miR-200 family (miR-200a, miR-200b, miR-200c) and miR-205 were obviously downregulated in cells that underwent EMT following TGF*β* treatment. Further studies revealed that these microRNAs could control E-cadherin expression via targeting SIP1 and ZEB1. Inhibition of these microRNAs was sufficient to induce EMT process in breast and prostate cancer cells [96, 97]. Xiao and colleagues reported that the expression of miR-200 family could be significantly enhanced by H_2_O_2_ treatment. The upregulation of miR-200-3p in turn modulated the H_2_O_2_-mediated oxidative stress response by targeting p38a [98]. Chen and colleagues investigated the miR-373 could promote EMT and tumor invasion in breast cancer by suppressing the expression of thioredoxin-interacting protein (TXNIP). Mechanistically, miR-373 activated the HIF1*α*-TWIST axis via activating TXNIP signaling. TWIST could induce miR-373 expression by binding to the promoter of the miR-371-373 cluster. Their study implied that miR-373 could induce tumor cell EMT and metastasis via miR-373-TXNIP-HIF1*α*-TWIST signaling axis in breast cancer [99]. Besides, Martello and colleagues identified miR-103/107 family, which attenuated microRNA biosynthesis by targeting Dicer, were closely related to breast cancer metastasis. Functionally, miR-103/107 confer migratory capacities and empower metastatic through regulating EMT process. Inhibition of miR-103/107 family could suppress cancer cell migration and metastasis [100]. These findings indicated the critical role of ROS-microRNA network in the regulation of tumor invasion and metastasis.

## 5. Conclusions

Collectively, growing studies have demonstrated microRNAs and oxidative stress interacted synergistically or antagonistically in the occurrence and progression of cancer. Under oxidative stress, ROS could regulate the biogenesis and expression of several microRNAs. At the same time, abnormally expressed microRNAs in turn inhibit or enhance the ROS generation by targeting multiple signaling pathways. Although the information on the interplay between oxidative stress and microRNA regulation is minimal, the complex crosstalk still provides both challenges and opportunities for the development of novel anticancer treatment. An ideal strategy for clinical application requires not only potent efficiency and specificity but also functional safety and bioactivity, as well as less toxicity. Further study on the microRNAs-oxidative stress network has a great possibility to develop novel anticancer therapeutic strategies, as microRNAs can be employed to regulate ROS-related cell proliferation and apoptosis or reduce ROS-mediated oxidative stress.

## Figures and Tables

**Table 1:** MicroRNA targets multiple ROS signaling pathways in cancer.

MicroRNAs	Targets	Mechanism	Relevant cancer	Ref.
miR-28	Nrf2/Keap1 pathway	Nrf2 downregulation; colony formation	Breast cancer	[24]
miR-200a	Nrf2/Keap1 pathway	Keap1 downregulation; Nrf2 activation	Breast cancer; hepatocellular carcinoma	[25, 101, 102]
miR-144	Nrf2/Keap1 pathway	Regulate the cisplatin resistance of lung cancer cells via Nrf2	Lung cancer	[103]
miR-93	Nrf2/Keap1 pathway	Downregulating Nrf2 and Nrf2-related genes	Breast cancer	[23]
miR-155	Nrf2/Keap1 pathway	Bach1 downregulation; upregulation of HO-1; enhance resistance to oxidative stress	Renal cancer; lung cancer	[26, 104, 105]
miR-125b	Nrf2/Keap1 pathway	Targeting the anti-oxidative gene PRXL2A	Oral squamous cell carcinoma	[27]
miR-29	Mitochondrial pathway	Promoting apoptosis through a mitochondrial pathway that involves Mcl-1 and Bcl-2	Hepatocellular carcinoma	[106]
miR-34a	Mitochondrial pathway	Inhibiting tumor growth and inducing apoptosis	Multiple myeloma	[33]
miR-30a	Mitochondrial pathway	Downregulating Beclin 1 and ATG5 expression; inhibiting autophagy	Chronic myeloid leukemia	[107]
miR-122	Mitochondrial pathway	Targeting mitochondrial metabolic genes	Hepatocellular carcinoma	[108]
miR-375	Mitochondrial pathway	Reducing expression of ATG7; inhibiting autophagy	Hepatocellular carcinoma	[109]
miR-450a	Mitochondrial pathway	Suppressing multiple genes involved in the EMT; targeting glutaminolysis related genes; reducing tumor migration and invasion	Ovarian cancer	[34]
miR-27b	Mitochondrial pathway	Suppressing PDHX; reducing mitochondrial oxidation and promoting extracellular acidification; promoting cell proliferation	Breast cancer	[110]
miR-31	Mitochondrial pathway	Inhibiting SIRT3; promoting tumor migration and invasion	Oral carcinoma	[36]
mitomiR-2392	Mitochondrial pathway	Reprogramming metabolism by downregulation of oxidative phosphorylation and upregulation of glycolysis; regulating chemoresistance	Tongue squamous cell carcinoma	[37]
miR-17-3p	Mitochondrial pathway	Inhibiting Mn-SOD, Gpx2, and TrxR2; enhancing radiosensitivity	Prostate cancer	[38]
miR-128a	Mitochondrial pathway	Bmi-1 downregulation; maintain mitochondrial function and reduce ROS generation	Cancer	[111]
miR-210	Mitochondrial pathway	ISCU downregulation; leading to upregulation of mitochondrial complex I activity and upregulation of mitochondrial ROS production	Cancer	[30, 112]
miR-663	Mitochondrial pathway	Targeting BBC3 and BTG2; regulating apoptosis by controlling MOMP	Lung cancer	[113]
miR-3174	Mitochondrial pathway	Targeting ARHGAP10; inhibiting mitochondria-dependent apoptosis and autophagy	Gastric cancer	[114]
miR-148a-3p	Mitochondrial pathway	Promoting mitochondrial fission and decreasing AKAP1 expression; sensitizing cisplatin treatment; suppressing RAB12 expression and mTOR1 activation	Gastric cancer	[115]
miR-485-3p, miR-485-5p	Mitochondrial pathway	Targeting and inhibiting the expression of PPARGC1A	Breast cancer	[116]
miR-195	Mitochondrial pathway	Targeting ACACA, FASN, HMGCR, and CYP27B1; inhibiting proliferation, invasion and migration; decreasing mesenchymal markers expression and enhancing epithelial markers	Breast cancer	[117]
miR-21	SOD/catalase pathway	Inhibiting T-AOC, PDCD4, SOD, and catalase	Gastric cancer	[118]
miR-146a	SOD/catalase pathway	Downregulating SOD2 and enhancing ROS generation; increasing apoptosis, inhibiting cell proliferation	Ovarian cancer; lung cancer	[47, 119]
miR-155	SOD/catalase pathway	Increasing the level of SOD2 and catalase through inhibiting DCK; causing chemoresistance	Pancreatic cancer	[48]
miR-212	SOD/catalase pathway	Targeting Mn-SOD; suppressing of Mn-SOD-induced metastasis	Colorectal cancer	[49]
miR-592	HIF-1*α* pathway	miR-592/WSB1/HIF-1*α* axis inhibiting glycolytic metabolism	Hepatocellular carcinoma	[120]
miR-199a-5p	HIF-1*α*/COX-2 pathway	Regulating tumor growth and angiogenesis	Cancer	[121]
miR-135b	HIF-1*α* pathway	Promoting cancer cell proliferation, colony formation, survival, and angiogenesis through activation of HIF-1*α*	Head and neck squamous cell carcinoma	[122]
miR-138	HIF-1*α* pathway	Suppressing cancer cell invasion and metastasis by targeting SOX4 and HIF-1*α*	Ovarian cancer	[123]
miR-186	HIF-1*α* pathway	Inhibiting aerobic glycolysis	Gastric cancer	[124]
miR-206	14-3-3*ζ*/STAT3/HIF-1*α*/VEGF pathway	Decreasing the angiogenesis by targeting 14-3-3*ζ* and inhibiting the STAT3/HIF-1*α*/VEGF pathway	Lung cancer	[125]

Nrf2: nuclear factor E2-related factor 2; Keap1: Kelch-like ECH-associated protein 1; HO-1: heme oxygenase-1; PRXL2A: peroxiredoxin-like 2A; ATG-5: autophagy-related 5; PDHX: pyruvate dehydrogenase complex component X; Mn-SOD: manganese superoxide dismutase; Gpx2: glutathione peroxidase 2; TrxR2: thioredoxin reductase 2; ISCU: iron-sulfur cluster assembly enzyme; BBC3: BCL2-binding component 3; BTG2: BTG antiproliferation factor 2; MOMP: mitochondrial outer membrane permeabilization; ARHGAP10: Rho GTPase-activating protein 10; AKAP1: A-kinase anchoring protein 1; PPARGC1A: PPARG coactivator 1 alpha; ACACA: acetyl-CoA carboxylase alpha; FASN: fatty acid synthase; HMGCR: 3-hydroxy-3-methylglutaryl-CoA reductase; CYP27B1: cytochrome P450 family 27 subfamily B member 1; T-AOC: total antioxidation competence; SOD: superoxide dismutase; CAT: catalase; PDCD4: programmed cell death 4 protein; DCK: deoxycytidine kinase; WSB1: WD repeat and SOCS box containing 1; STAT3: signal transducer and activator of transcription 3; VEGF: vascular endothelial growth factor.

**Table 2:** Summary of microRNAs regulated by the transcription factors and proteins under oxidative stress in carcinogenesis.

Transcription factors/proteins	Target microRNAs	Mechanism	Relevant cancer	Ref.
NF-*κ*B	miR-19a	Suppressing cell proliferation; regulating apoptosis	Colorectal cancer	[63, 126]
NF-*κ*B	miR-21	Promoting cell proliferation and cancer metastasis; suppressing cell apoptosis	Lung cancer; colorectal cancer; myeloma	[60, 127–130]
NF-*κ*B	miR-148a and miR-152	Modulating tumor angiogenesis and cancer progression	Breast cancer	[131]
NF-*κ*B	miR-155	Promoting cell proliferation	Breast cancer	[129]
NF-*κ*B	miR-489	KRAS/NF-*κ*B/YY1/miR-489 axis regulating tumor migration and metastasis	Pancreatic cancer	[132]
NF-*κ*B	miR-29	NF-*κ*B/YY1/miR-29 axis regulating tumor growth and cell differentiation	Rhabdomyosarcoma	[133]
NF-*κ*B	miR-29c	Regulating MMP-9 expression, secretion and activation; inhibiting tumor invasiveness	Pancreatic cancer	[134]
NF-*κ*B	miR-425	Regulating apoptosis via miR-425/PTEN axis	Breast cancer	[135]
HIF-1*α*	miR-210	HIF-1*α*/miR-210/HIF-3*α* inhibiting TIMP2; promoting metastasis	Hepatocellular carcinoma	[136]
HIF-1*α*	miR-212	Promoting progression	Pancreatic cancer	[137]
HIF-1*α*	miR-26 and miR-29	Regulating tumor metastasis	Hepatocellular carcinoma	[138]
HIF-1*α*	miR-23a∼27a∼24 cluster	Promoting cancer progression via reprogramming metabolism	Colorectal cancer	[139, 140]
HIF-1*α*	miR-200b	Modulating the EMT	Colorectal cancer	[141]
HIF-1*α*	miR-382	Promoting angiogenesis and acting as an angiogenic oncogene by repressing PTEN	Gastric cancer	[68]
HIF-1*α*	miR-646	HIF-1*α*/miR-646/MIIP axis contributing to tumor progression	Pancreatic cancer	[142]
HIF-1*α*	miR-224	Promoting cell growth, migration and invasion by targeting RASSF8	Gastric cancer	[143]
HIF-1*α*	miR-145	Regulating apoptosis	Bladder cancer	[144]
HIF1*α*/HDAC1	miR-548an	Inhibiting miR-548an expression, resulting in the upregulation of vimentin that facilitates the tumorigenesis	Pancreatic cancer	[145]
HIF-1*α*/HIF-2*α*	miR-210	Regulating tumor progression	Cholangiocarcinoma; bladder cancer	[146, 147]
C-Myc	miR-137	Regulating c-Myc-EZH2 axis; regulating cisplatin resistance	Ovarian cancer	[71]
C-Myc	miR-17-92	Maintaining a neoplastic state by suppressing specific target genes	Cancer	[72]
Myc/EZH2/ HDAC3	miR-29, miR-494	Regulating lymphoma growth	Lymphoma	[75]
HDAC4	miR-1, miR-206	Promoting cancer progression	Lung cancer	[73]
HDACs	miR-15a, miR-16, and miR-29b	Silencing miR-15a, miR-16, and miR-29b	Chronic lymphocytic leukemia	[148]
SAHA	miR-129-5p	Inducing cancer cell apoptosis	Lung cancer	[74]
p53	miR-19a	Promoting myeloma cells invasion by upregulating miR19a/CXCR5	Multiple myeloma	[149]
p53	miR-34a, miR-16	Targeting Bcl2 to induce apoptosis	Lung cancer	[150]
p53	miR-605	Targeting and repressing PSMD10 expression, inhibiting cancer progression	Cholangiocarcinoma	[151]
p53	miR-192-5p, miR-215	Promoting apoptosis by activating the p53-miR-192-5p/215-XIAP pathway; inducing cell cycle arrest	Lung cancer	[152, 153]
p53	miR-107	Inhibiting HIF-1 and tumor angiogenesis	Colorectal cancer	[154]
p53	miR-16-2	Inducing cell cycle arrest and apoptosis	Hepatocellular carcinoma	[155]
Ferroportin	miR-17-5p	Promotes cell proliferation by modulating the Nrf2-miR-17-5p axis	Multiple myeloma	[156]
Kallistatin	miR-21, miR-34a	inhibiting TGF*β*-induced endothelial-mesenchymal transition by differential regulation of microRNA-21 and eNOS expression	Breast cancer	[157, 158]
Curcumin	miR-27a, miR-20a, and miR-17-5p	Inhibiting cell growth and inducing apoptosis via inducing ROS; decreasing specificity protein transcription factors by targeting microRNAs	Colon cancer	[159]

NF-*κ*B: nuclear factor kappa-B; YY1: YY1 transcription factor; MMP9: matrix metallopeptidase 9; HIF-1*α*: hypoxia-inducible factor 1, alpha subunit; TIMP2: TIMP metallopeptidase inhibitor 2; PTEN: phosphatase and tensin homolog; MIIP: migration and invasion inhibitory protein; SAHA: suberoylanilide hydroxamic acid; RASSF8: Ras association domain family member 8; EZH2: enhancer of zeste 2 polycomb repressive complex 2 subunit; CXCR5: C-X-C motif chemokine receptor 5; PSMD10: proteasome 26S subunit, non-ATPase 10; eNOS: endothelial nitric oxide synthase; XIAP: X-linked inhibitor of apoptosis.
